# Intake levels and main sources of nutrients for Japanese children with motor or intellectual disabilities

**DOI:** 10.1017/jns.2023.108

**Published:** 2023-12-21

**Authors:** Toshiko Takezoe, Kentaro Murakami, Jun Fujishiro, Miwako Horikawa, Mitsuru Kubota, Yutaka Kanamori, Nagahisa Takahashi, Yuko Nakano, Misato Migita, Hiroshi Matsufuji, Satoshi Sasaki

**Affiliations:** 1 Department of Pediatric Surgery, The University of Tokyo Hospital, Tokyo, Japan; 2 Department of Social and Preventive Epidemiology, School of Public Health, The University of Tokyo, Tokyo, Japan; 3 Department of General Pediatrics & Interdisciplinary Medicine, National Center for Child Health and Development, Tokyo, Japan; 4 Division of Surgery, Department of Surgical Specialties, National Center for Child Health and Development, Tokyo, Japan; 5 Department of Pediatrics, National Rehabilitation Center for Children with Disabilities, Tokyo, Japan; 6 Department of Pediatrics, Ashikaga Hospital, Tochigi, Japan; 7 Department of Pediatric Surgery, St. Luke's International Hospitals, Tokyo, Japan

**Keywords:** Children with disabilities, Dietary intakes, Dietary record, Feeding difficulties, Food sources, Intellectual disability, Malnutrition, Motor disability

## Abstract

Proper nutritional management is important for the growth and development of children with motor or intellectual disabilities; however, few studies have investigated the nutrient intake of children with disabilities. This study aimed to investigate the nutrient intake and food groups that are the main sources of nutrients for children with disabilities. This cross-sectional observational study included twenty-five children (mean age, 11⋅8 years) from five hospitals in Japan. Using a 3-d weighed dietary record, we estimated the daily nutrient intake and food and beverage sources that contributed to nutrient intake. The mean values of calcium, magnesium, iron, vitamin A, thiamine, riboflavin, and vitamin C intake were below the recommended dietary allowance, and those of dietary fiber and potassium were below the levels recommended by the Tentative Dietary Goal for Preventing Lifestyle-related Diseases (DG). In contrast, the mean intake values of fat, saturated fatty acids, and sodium were above the DG levels. Dairy products, meat, vegetables, and cereals were found to be the major contributors of nutrients. Increased intake of vegetables may help alleviate insufficient micronutrient intake in children with disabilities.

## Introduction

Adequate nutrient intake is not only important for normal growth and development in childhood but also important for preventing the onset of diseases in adulthood. For example, lifestyle-related diseases, including cardiovascular disease in adulthood, are not just a result of classic risk factors (such as blood pressure and smoking) that appear in mid-life but also of dietary exposures across the life course.^(1)^ Additionally, dietary patterns established during childhood are significant determinants of the extent of the risk of cardiovascular disease in adulthood.^(2)^ Furthermore, toddlers and adolescents are more likely to suffer from poor nutrition than adults because of the high nutritional demands for growth and development.^(3)^ Providing adequate nutrition to these populations is important to ensure maximal physical and cognitive development; this is also true for children with disabilities.^(4–7)^ One study mentioned that self-feeding skills were highly related to life expectancy, suggesting that the feeding function may be as important as mental and motor capacity as an indicator of the health outcomes of children with disabilities.^(8)^

Particularly for children with disabilities, nutritional assessment and support are essential aspects of routine and medical care.^(3)^ Feeding and swallowing difficulties in children with severe disabilities often cause complications that severely affect nutrient intake and lead to malnutrition, dehydration, alterations in linear growth, and micronutrient deficiency.^(3,8)^ Children with intellectual disabilities, such as autism spectrum disorder, are likely to be at risk of inadequate intake of minerals and vitamins because of their preferences for specific food textures, colors, and flavors.^(9,10)^ Adequate nutritional support may restore linear growth, decrease irritability, reduce the frequency of hospitalization, and increase social participation.^(3,11,12)^

Many studies have reported inadequate intake of micronutrients, such as calcium, iron, and zinc in children with disabilities in Western and Middle Eastern countries.^(3,6,9,11,13–18)^ Only one report described the nutrient intake of children with disabilities in Japan and reported inadequate intake of calcium, zinc, and iodine.^(19)^ It has been suggested that inadequate intake of several nutrients in children with disabilities is primarily caused by energy deficits due to complications such as gastroesophageal reflux and dysphagia.^(6,8,20)^ However, the overconsumption or underconsumption of certain foods is also considered a cause of nutrient deficiency. For example, Sullivan *et al.* suggested that high milk intake leads to iron deficiency because high milk intake satisfies the appetite and reduces the intake of other foods.^(6)^ Conversely, Hasegawa *et al.* suggested that a delay in switching from standard infant formula to enteral formula adversely affected lipid metabolism and that inadequate intake of selenium and iodine was observed in children on nutritional supplements with low trace element content.^(19)^

Thus, information on the source of each nutrient can help physicians address the excess and deficiency of nutrient intake. However, to our knowledge, no study has examined the contribution of food sources to the intake of specific nutrients in children with disabilities. Therefore, this study aimed to investigate the food groups that are the main sources of nutrients for Japanese children with motor or intellectual disabilities who require assistance with food intake.

## Materials and methods

### Study design and sample

This cross-sectional study was conducted between 22 October 2020 and 28 February 2021 in five hospitals (University of Tokyo Hospital, National Rehabilitation Center for Children with Disabilities, St Luke's International Hospital, Ashikaga Hospital, and National Center for Child Health and Development). The inclusion criteria were as follows: (1) age, 6–17 years; (2) presence of motor or intellectual disabilities and self-feeding impairment; (3) living at home or in an institution; (4) oral intake of food or tube feeding via gastrostomy; and (5) assumed to be receiving about half or more of the energy intake from food (as reported by their parents). We excluded: (1) children with infection requiring systemic antibiotic therapy or admission to the infirmary (a nursing ward for intensive patient observation) within the time frame for physical measurements, i.e. the start of the survey; (2) children with malignancies; and (3) children who regularly used medications known to affect growth or body composition (e.g. steroids, thyroxine).

This study was conducted in accordance with the guidelines of the Declaration of Helsinki, and the Ethics Committee of the University of Tokyo Faculty of Medicine approved all the procedures involving human subjects (2020218NI). Written informed consent was obtained from the parents of all the participants.

### Measures

#### Demographics, anthropometric measurements, and clinical factors

Data on the age and sex of the children were obtained on the days of outpatient visits. Measurements of body weight and height were conducted at the same time, using scales available in each hospital. Participants who could not stand on their own were weighed together with a parent, and the parent's weight was subtracted. The body height of participants who could stand was measured in the standing position. The height of those who could not stand on their own was expressed by measuring three sections (the division method).^(21)^ The body mass index (kg/m^2^) was calculated using the weight and height measurements. The subscapular and triceps skinfold thicknesses were measured twice using a skinfold digital scale (SR-803; Tanita, Tokyo, Japan). The average of two measurements was used to estimate body fat percentage, based on the original and corrected Slaughter equations for children with cerebral palsy.^(22)^

Using a specially designed questionnaire (completed by the participants’ parents), we collected data on diagnosis, disability characteristics, puberty onset, and gross motor function classification system (GMFCS). The GMFCS is a system that classifies gross motor function in children with cerebral palsy.^(23)^ Although this system was developed for children with cerebral palsy, it has been used for children with neurological disabilities other than cerebral palsy.^(3,24)^ The degree of intellectual disability was not measured, but the response of ‘No’ to any of the following questions was considered to reflect some kind of intellectual disability, e.g. ‘Whether or not they can eat with no assistance?’ or ‘Whether or not they can report their appetite?’.

The participants were divided into two groups according to the GMFCS level: mild motor impairment group (GMFCS levels 1–3) and severe motor impairment group (GMFCS levels 4 and 5). Owing to the small number of participants, the values were not compared by statistical analysis.

#### Dietary assessment

Dietary data were collected using a three-nonconsecutive-day weighed dietary record, which included two weekdays with lunch at school and one weekend day (without lunch at school). In this study, the school dietitians and teachers collected the dietary data from the schools, whereas the parents collected the dietary data from outside the schools.

Each participant's parent was asked to record the food and beverages consumed outside the school. Each parent was given an oral explanation of the diet-recording method, digital kitchen scale (KJ-212; Tanita, Tokyo, Japan), measuring spoon, measuring cup, booklet for the dietary record, and recording sheets. The parents were asked to weigh and record all food and beverage items consumed by the participants (as far back as possible to the ingredient level), in addition to any leftovers and spills. When weighing was difficult (e.g. dining out, taking away, or eating ready-made food), the parents were instructed to document as much information as possible, including the brand name of the food and the consumed portion size (based on typical household measures) as well as the details of the leftovers. The parents were also asked to describe the food/fluid textures using a scale that was specially developed for this study for each dish. This scale was based on the dysphagia diet classification for children/people with dysphagia 2018 by the Japanese Society of Dysphagia Rehabilitation^(25)^ and consisted of five levels for rice (0: standard rice, 1: soft rice, 2: rice porridge, 3: jelly shaped rice porridge, 4: pureed rice porridge), four levels for fluids (beverages and soups) (0: not thick, 1: thick enough to drink quickly with a straw, 2: thick enough to be sucked uncomfortably through a straw, 3: thick enough to not be sucked through a straw), and five levels for other dishes (0: standard, 1: soft, 2: mashed, 3: mousse-like, 4: pureed). Within a few days after the recording day, the parents were asked to send photographs of the recording form and empty packages of any processed foods consumed to the researcher. The researcher checked the photographed forms and, whenever necessary, sought additional information via an online call.

School dietitians/teachers recorded the foods and beverages consumed by the participants at school. The participants’ parents provided the school dietitians/teachers with the recording sheets during their school stay. We also asked the school dietitians/teachers to weigh and record all foods and beverages the participants consumed at school on the recording days, in addition to any leftovers or spills. They were asked to describe the food/fluid textures of each dish using the aforementioned scales. Finally, the school dietitians/teachers handed the forms to the parents, and the parents mailed them to the researcher. The school dietitians and teachers were interviewed about missing information by the researcher via the parents, whenever necessary.

The data recorded in the recording forms were entered into an Excel spreadsheet specially designed for this study. Then, the estimates of portion sizes recorded using household measures were converted into weights, and all individual food items were coded using the Standard Tables of Food Composition in Japan.^(26)^ A few food items not included in the tables were assigned nutrient values of similar food items (e.g. beet sugar). For some processed foods (e.g. interventional food), the weight of each ingredient was determined using the ingredient names and nutritional information labels in the product information. We calculated the estimates of daily energy intake and selected nutrients using Standard Tables of Food Composition in Japan.^(26)^ Although some participants took magnesium preparations as laxatives, we did not include nutrient intake from medications because we intended to estimate the nutrient intake only from foods and enteral formulas.

#### Assessment of nutrient intake adequacy

To assess nutrient intake adequacy, we estimated the daily nutrient intake per individual. We compared the daily nutrient intake with age- and sex-specific reference values for each individual, according to the Dietary Reference Intakes for Japanese (DRIs)^(27)^ (Supplementary Tables S1 and S2) and computed the percentage of participants whose intake levels did not meet the DRIs. Of the total 33 nutrients presented in the DRIs, we excluded three nutrients (biotin, chromium, and molybdenum) from the present analysis because of insufficient food composition data for them.^(27)^ The DRIs are based on the Recommended Dietary Allowance — ‘an intake level that exceeds the requirements of 97–98% of all individuals when requirements in the group have a normal distribution’.^(28)^ For evaluating nutrients for which no RDA values were established, the tentative dietary goals for preventing lifestyle-related diseases (DG) were used. The DGs are ‘the average daily nutrient intake levels (or ranges) that the Japanese should currently aim to consume primarily to prevent chronic diseases’.^(27)^ The Japanese DRIs also have tolerable upper intake levels (ULs) for several nutrients. We calculated the percentage of participants who consumed nutrients at levels below the RDA (for protein, calcium, magnesium, iron, zinc, copper, iodine, selenium, vitamin A, thiamine, riboflavin, niacin, vitamin B6, vitamin B12, folate, and vitamin C), levels greater than or less than the DG (for fat, saturated fatty acid [SFA], carbohydrate, dietary fiber, sodium, and potassium), and levels greater than the ULs (for iron, iodine, selenium, vitamin A, vitamin D, vitamin E, niacin, vitamin B6, and folate) for assessing the inadequacy of nutrient intake.

#### Assessment of food sources for each nutrient

We calculated the contribution of each food source (%) to the total nutrient intake of all participants, energy requirements, and adequacy of the thirty nutrients listed in the DRIs. The calculations were based on the intake of all foods and beverages (excluding enteral formula). The food groups were defined and classified according to the classification in the Standard Tables of Food Composition in Japan 2015 and 2018 Addendum.^(26)^

## Results

This analysis included twenty-five children aged 7–16 years (Table 1). Fifteen children (60 %) had severe motor disabilities, and ten children (40 %) had mild motor disabilities. For all body measurements, the values of the participants with severe motor disabilities were lower than those of the participants with mild motor disabilities. Similarly, for energy intake, the former group had a lower mean energy intake than did the latter. All participants took some or all of their diet orally, and more than half required the food to be texturally adapted because of dysphagia.

**Table 1.** Basic characteristics and anthropometric measures of study participants according to gross motor functional ability (*n* 25)

		All children (*n* 25)	GMFCS category	
			I, II, III (*n* 10)	IV, V (*n* 15)
Sex; *n* (%)	Male	12 (48 %)	7 (70 %)	6 (40 %)
	Female	13 (52 %)	3 (30 %)	9 (60 %)
Age (years); mean ± sd		11⋅8 ± 3⋅1	11⋅4 ± 3⋅5	12⋅1 ± 2⋅8
EI (J/d); mean ± sd^a^		6674 ± 2428	7980 ± 2567	5803 ± 1876
Anthropometric measures				
Height (cm); mean ± sd		132⋅3 ± 20⋅4	138⋅9 ± 20⋅9	128⋅2 ± 19⋅6
Weight (kg); mean ± sd		30⋅0 ± 15⋅7	41⋅0 ± 19⋅2	23⋅2 ± 7⋅7
BMI; mean ± sd		16⋅3 ± 4⋅4	20⋅0 ± 4⋅2	13⋅9 ± 2⋅5
Triceps skinfold (cm); mean ± sd		0⋅9 ± 0⋅3	1⋅0 ± 0⋅4	0⋅8 ± 0⋅3
Subscapular skinfold (cm); mean ± sd		0⋅8 ± 0⋅4	1⋅0 ± 0⋅5	0⋅7 ± 0⋅2
Body fat (percentage weight); mean ± sd		15⋅9 ± 3⋅2	16⋅6 ± 3⋅7	15⋅4 ± 2⋅7
Medical cares				
Ventilator-assisted^b^; *n* (%)	Yes	1 (4 %)	0 (0 %)	1 (7 %)
	No	24 (96 %)	10 (100 %)	14 (93 %)
Tube feeding^c^; *n* (%)	Yes	7 (28 %)	2 (20 %)	5 (33 %)
	No	18 (72 %)	8 (80 %)	10 (67 %)
Texture of food/fluid				
Thick liquid foods^d^; *n* (%)	Yes	11 (44 %)	1 (10 %)	10 (67 %)
	No	14 (56 %)	9 (90 %)	5 (33 %)
Texture of food; *n* (%)	Normal	11 (44 %)	8 (80 %)	3 (20 %)
	soft rice, rice porridge, soft or mashed food	5 (20 %)	1 (10 %)	4 (27 %)
	jelly-like or pureed rice porridge, mousse-like or pureed food	9 (36 %)	1 (10 %)	8 (53 %)

GMFCS, gross motor functional classification system; EI, energy intake; BMI, body mass index.

a EI calculated as a 3-d mean per participant was used.

b Ventilator-assisted: Yes = both partial and exclusive ventilator-assisted; No = no respiratory assistance required.

c Tube feeding: Yes = partial tube feeding; No = exclusive oral feeding.

d Thick liquid foods; Yes = beverages or liquid foods thickened with starch/thickener due to dysphagia; No = not thickened, regardless of dysphagia.

The mean values of daily energy and nutrient intake are listed in Table 2. The mean daily intake levels of protein, copper, iodine, selenium, vitamin B12, folate, and vitamin C were higher than the RDAs for almost all age groups for both boys and girls. In contrast, the mean daily intake of calcium of all participants was less than the RDA at the age of 6–7 years. Further, more than half of the participants had intake values below the RDA for calcium, magnesium, iron, vitamin A, thiamine, riboflavin, and vitamin C. The mean values of daily intake of fat, SFA, and sodium exceeded the DGs in 60, 52, and 44 % of the participants, respectively. In contrast, the mean values of daily intake of dietary fiber and potassium were below the DGs in 76 and 56 % of the participants, respectively. In addition, iodine and vitamin A values were above the ULs in 12 % of participants who took iodine and 8 % who took vitamin A supplements.

**Table 2.** Energy and nutrient intakes from foods, beverages, and formulas (*n* 25)^a^

	Mean	SD	DRI compliance (%)^b^			
			<RDA	<DG	>DG	>UL
**Macronutrients**						
Protein (g/d)	57⋅7	19⋅4	44	−	−	−
Fat (g/d)	55⋅3	21⋅0	−	0	60	−
SFA (g/d)	18⋅5	9⋅0	−	−	52	−
*n*-6PUFA (g/d)	8⋅6	3⋅9	−	−	−	−
*n*-3PUFA (g/d)	1⋅6	0⋅7	−	−	−	−
Carbohydrate (g/d)	213⋅7	88⋅4	−	28	4	−
Dietary fiber (g/d)	10⋅6	5⋅2	−	76	−	−
**Micronutrients**						
Sodium (g NaCl equivalent/d)	6⋅6	3⋅4	−	−	44	−
Potassium (mg/d)	2158⋅7	747⋅3	−	56	−	−
Calcium (mg/d)	598⋅2	181⋅3	80	−	−	−
Magnesium (mg/d)	208⋅0	76⋅6	64	−	−	−
Phosphorus (mg/d)	919⋅2	268⋅4	−	−	−	−
Iron (mg/d)	6⋅9	3⋅3	88	−	−	0
Zinc (mg/d)	8⋅0	2⋅8	48	−	−	−
Copper (mg/d)	0⋅9	0⋅4	28	−	−	−
Manganese (mg/d)	2⋅0	0⋅9	−	−	−	−
Iodine (μg/d)	499⋅2	536⋅9	32	−	−	12
Selenium (μg/d)	49⋅3	20⋅1	4	−	−	0
Vitamin A (μg RAE/d)	701⋅2	551⋅9	72	−	−	8
Vitamin D (μg/d)	6⋅2	5⋅0	−	−	−	0
Vitamin E (mg/d)	8⋅7	5⋅8	−	−	−	0
Vitamin K (μg/d)	180⋅6	154⋅3	−	−	−	−
Thiamine (mg/d)	0⋅9	0⋅4	80	−	−	−
Riboflavin (mg/d)	1⋅3	0⋅4	68	−	−	−
Niacin (mg/day)	14⋅0	5⋅5	48	−	−	0
Vitamin B6 (mg/d)	1⋅3	0⋅6	36	−	−	0
Vitamin B12 (μg/d)	4⋅5	2⋅4	12	−	−	−
Folate (μg/d)	256⋅5	110⋅4	40	−	−	0
Pantothenic acid (mg/d)	5⋅8	2⋅0	−	−	−	−
Vitamin C (mg/d)	106⋅7	70⋅4	52	−	−	−

DRI, dietary reference intake; RDA, recommended dietary allowances; DG, tentative dietary goal for preventing lifestyle-related diseases; UL, tolerable upper intake level; SFA, saturated fatty acid; PUFA, polyunsaturated fatty acid.

a Energy and nutrient intakes were estimated as the average of the 3-d weighed dietary-record data. Medicines and supplements were not included.

b DRI compliance indicates the percentage of subjects above or below each reference value. When comparing to each reference value,^(27)^ only age and sex were matched and not energy levels.

The top three food sources for each nutrient are presented in Table 3. Dairy products and meat (beef, pork, chicken, and processed meat) were among the top three contributors, accounting for nineteen and eighteen of the thirty-one nutrients, respectively. Vegetables and cereals were the second major contributors. The top three food sources accounted for more than 80 % of the intakes of iodine, vitamin A, vitamin D, vitamin K, vitamin B12, and vitamin C, whereas the contribution of the top three food groups was relatively small (less than 50 %) for potassium, magnesium, iron, and vitamin E.

**Table 3.** Contribution (%) of food sources to energy and nutrient intakes from foods and beverages of study participants (*n* 25)^a^

	Major food sources^b^					
	1st		2nd		3^rd^	
Energy	Cereals	30⋅2	Dairy products	13⋅5	Meats	11⋅3
**Macronutrients**						
Protein	Meats	24⋅5	Dairy products	16⋅8	Cereals	16⋅4
Fat	Meats	23⋅1	Dairy products	20⋅2	Fats and oils	19⋅5
SFA	Dairy products	33⋅0	Meats	23⋅4	Fats and oils	17⋅8
*n*-6PUFA	Fats and oils	24⋅6	Pulses	17⋅2	Meats	13⋅8
*n*-3PUFA	Fish, shellfish, sea products	31⋅8	Fats and oils	14⋅2	Pulses	12⋅9
Carbohydrate	Cereals	47⋅5	Dairy products	8⋅8	Sugars and sweeteners	7⋅6
Dietary fiber	Vegetables	37⋅6	Cereals	15⋅6	Algae	8⋅9
**Micronutrients**						
Sodium	Seasonings and spices	64⋅5	Cereals	6⋅5	Dairy products	5⋅7
Potassium	Vegetables	21⋅8	Dairy products	17⋅6	Meats	10⋅4
Calcium	Dairy products	54⋅8	Vegetables	10⋅7	Pulses	6⋅4
Magnesium	Pulses	15⋅6	Vegetables	14⋅3	Dairy products	14⋅0
Phosphorus	Dairy products	28⋅0	Meats	15⋅0	Cereals	12⋅6
Iron	Vegetables	13⋅7	Meats	12⋅8	Pulses	12⋅0
Zinc	Meats	25⋅0	Cereals	22⋅1	Dairy products	16⋅2
Copper	Cereals	30⋅1	Pulses	13⋅3	Vegetables	12⋅4
Manganese	Cereals	48⋅2	Vegetables	15⋅4	Pulses	7⋅5
Iodine	Seasonings and spices	75⋅6	Algae	13⋅1	Dairy products	6⋅7
Selenium	Cereals	19⋅1	Eggs	18⋅6	Meats	18⋅2
Vitamin A	Vegetables	40⋅8	Meats	24⋅5	Dairy products	18⋅4
Vitamin D	Fish, shellfish, sea products	59⋅6	Dairy products	21⋅0	Eggs	8⋅9
Vitamin E	Vegetables	22⋅8	Fats and oils	16⋅5	Dairy products	10⋅6
Vitamin K	Vegetables	47⋅0	Pulses	33⋅0	Meats	4⋅9
Thiamine	Meats	33⋅6	Dairy products	13⋅5	Vegetables	11⋅7
Riboflavin	Dairy products	35⋅2	Meats	14⋅8	Eggs	10⋅1
Niacin	Meats	34⋅2	Fish, shellfish, sea products	15⋅4	Seasonings and spices	8⋅5
Vitamin B6	Meats	20⋅5	Vegetables	18⋅5	Beverages	11⋅5
Vitamin B12	Fish, shellfish, sea products	49⋅6	Meats	18⋅5	Dairy products	18⋅3
Folate	Vegetables	38⋅9	Fruits	11⋅5	Pulses	7⋅8
Pantothenic acid	Dairy products	23⋅6	Meats	15⋅8	Cereals	13⋅6
Vitamin C	Vegetables	42⋅6	Fruits	32⋅3	Potatoes and starches	7⋅6

SFA, saturated fatty acid; PUFA, polyunsaturated fatty acid.

a Contribution (%) of food sources was calculated from foods and beverages consumed by all participants (*n* 25) over a 3-d period.

b Food sources were defined based on the culinary usage and the similarity of nutrient profiles of the foods, mainly according to the Standard Tables of Food Composition in Japan 2015.^(26)^ The contribution of each food source to the total amount of each selected nutrient was calculated, and the top three food sources are shown.

## Discussion

In this study involving twenty-five children with disabilities, the mean intakes of iron, calcium, thiamine, vitamin A, riboflavin, magnesium, vitamin C, dietary fiber, and potassium were below the DRIs, whereas the mean intakes of total fat, SFAs, and sodium exceeded them. In addition, dairy products, meat, vegetables, and cereals were the major contributors of most nutrients. To our knowledge, this is the first study to examine the contribution of different food groups to the nutrient intake levels of children with disabilities.

Micronutrient deficiency is an important nutritional issue in children with motor and intellectual disabilities. Previous studies have shown that the intake levels of certain nutrients, namely vitamin D,^(3,9,11,14)^ calcium,^(3,9,15,16,19)^ iron,^(6,11,14,16)^ and zinc,^(3,6,19)^ are deficient in children with motor or intellectual disabilities. One report from Japan showed inadequate intake levels of iron, calcium, zinc, and iodine among children with severe motor and intellectual disabilities.^(19)^ In our study, insufficient micronutrient intake was observed. Some reports have suggested that one of the causes of inadequate micronutrient intake in children with disabilities is inadequate overall dietary intake.^(6,29)^ A study of healthy children aged 8–14 years in Japan reported that the intake levels of most vitamins and minerals were low,^(30)^ suggesting that insufficient intake of micronutrients in children with disabilities was not only due to quantitative adequacy. The cause of micronutrient deficiency is unclear, and it is suspected to be related to a combination of various factors, including deficiency due to gastroesophageal reflux, esophagitis,^(6)^ or the use of fistulas that are not age-appropriate.^(19)^ Further studies are necessary to describe the dietary intake of disabled children. However, the present study showed that the mean intake levels of total fat, SFAs, and sodium were excessive when assessed according to the DGs.

This finding is consistent with the excessive intake of fat and sodium reported in a previous study of healthy children aged 8–14 years in Japan.^(30)^ Fat intake among Japanese adults and children has been increasing,^(31)^ and the same is true for children with disabilities. Furthermore, in our study, the iodine levels in 12 % of the participants exceeded the ULs, and vitamin A levels exceeded the ULs in 8 %. It should be noted that both iodine and vitamin A are nutrients with large daily and interseasonal variations.^(32,33)^ Thus, it is necessary to re-examine the results after collecting long-term intake data in future research. In this study, mainly because we did not identify severe misreporting (underreporting) of dietary intake in our population, we used crude (rather than energy-adjusted) estimates of nutrient intakes to compare reference values derived from the Dietary Reference Intakes for Japanese 2020. We considered this procedure appropriate because we examined the inadequacy (or adequacy) of the nutrient intake of each participant relative to the reference value, which was sex- and age-specific.

Given the inadequate nutrient intake levels among children with disabilities, it is important to identify the principal food sources contributing to energy and nutrients. In our study, cereals (30⋅2 %) were the largest contributors to energy intake, followed by dairy products (13⋅5 %) and meat (11⋅3 %). In a study of healthy Japanese males and females aged 1 year and older, cereals (38⋅9 %), meat (15⋅1 %), and dairy products (5⋅5 %) contributed the most to energy. Our findings suggest that dairy products are important contributors to the energy requirement.^(34)^ Children with disabilities consume large amounts of dairy products because they are easy to chew and swallow, and the high dairy intake may satisfy their appetites and reduce their intake of other foods, which may cause iron intake deficiencies.^(6)^ In some countries, the major sources of energy for healthy adults and children are not necessarily the same food groups that provide rich sources of key micronutrients, causing inadequate intake of micronutrients.^(35–37)^ However, in the current study, the food groups contributing to energy (cereals, dairy products, and meat) and those contributing to all nutrients (dairy products, meat, vegetables, and cereals) were almost identical, indicating that the food groups are optimally balanced. The results also suggest that in order to address insufficient micronutrient intake, it is necessary to increase the intake of vegetables. The conclusion we arrived at regarding nutrient intake among Japanese children with motor or intellectual disabilities may not be groundbreaking, but we deem our findings significant as they constitute the first empirical evidence observed in this specific population.

Our study has several limitations, namely reporting errors and within-person variance in nutrient intake. Dietary recording methods are generally susceptible to measurement errors due to recording errors and potential changes in eating behavior,^(38)^ and it has been reported that dietary records of children with disabilities tend to overreport food intake.^(9)^ No study of children with disabilities has shown diurnal variations in diet, but in general, daily fluctuations in energy or nutrient intake are known to be intra-individual errors that occur due to daily differences in individual dietary intake, in addition to measurement errors in intake.^(39–41)^ The 3-d dietary record was too short to allow habitual intake to be estimated with the required accuracy; however, since the dietary recording method places a heavy burden on the participants, a period longer than 3 d was not considered feasible. Any dietary survey cannot be conducted without measurement error. Nevertheless, we conducted our dietary survey as accurately as possible, including a careful explanation of the purpose and procedure of this study to parents, resulting in the success of 3-d dietary record by all participants, interviews by trained dietitians after completing the dietary record, and a careful coding of foods during the development of dietary dataset. The small sample size, even compared with that of previous studies conducted in similar populations abroad, is also a major limitation of this study. Although we made every effort to include as many participants as possible, future research should be conducted on a larger sample of Japanese children with motor or intellectual disabilities to draw firm conclusions. The third limitation is the use of DRIs meant for apparently healthy individuals. Because most children with motor disabilities are smaller in stature than healthy children of the same sex and age, their intake values tend to be judged as being sufficient relative to the DRIs. However, since there are currently no reference values available for children with disabilities, we had no choice but to use the DRIs.

In conclusion, we found that the mean intake levels of several micronutrients (iron, calcium, thiamine, vitamin A, riboflavin, magnesium, vitamin C, and potassium) and dietary fiber were below the DRIs. The food groups contributing to energy intake (i.e. cereals, dairy products, and meat) also contributed to micronutrient intake. Vegetables are known to be sources of key micronutrients; therefore, increased vegetable intake may help alleviate insufficient micronutrient intake in children with disabilities.

## Supporting information

Takezoe et al. supplementary material 1 Takezoe et al. supplementary material

Takezoe et al. supplementary material 2 Takezoe et al. supplementary material

## Supplementary material

The supplementary material for this article can be found at https://doi.org/10.1017/jns.2023.108
