# Supplementary material for: Intake levels and main sources of nutrients for Japanese children with motor or intellectual disabilities
Source: J Nutr Sci. 2023 Dec 21;12:e126. doi: 10.1017/jns.2023.108 (PMC10753447; doi:10.1017/jns.2023.108)
Supplement: Takezoe et al. supplementary material 1 — Takezoe et al. supplementary material [file S2048679023001088sup001.pdf]

DRI Value（boys）[a]

Nutrients	Value														
	RDA					DG					Ul				
	Age（years）					Age（years）					Age（years）				
	6 - 7	8 - 9	10 - 11	12 - 14	15 - 17	6 - 7	8 - 9	10 - 11	12 - 14	15 - 17	6 - 7	8 - 9	10 - 11	12 - 14	15 - 17
Protein (g/day)	30	40	45	60	65	13 - 20	13 - 20	13 - 20	13 - 20	13 - 20	-	-	-	-	-
Fat (% energy)	-	-	-	-	-	13 - 20	13 - 20	13 - 20	13 - 20	13 - 20	-	-	-	-	-
SFA (% energy)	-	-	-	-	-	≤ 10	≤ 10	≤ 10	≤ 10	≤ 8	-	-	-	-	-
n-6PUFA (g/day)	-	-	-	-	-	-	-	-	-	-	-	-	-	-	-
n-3PUFA (g/day)	-	-	-	-	-	-	-	-	-	-	-	-	-	-	-
Carbohydrate (% energy)	-	-	-	-	-	50 - 65	50 - 65	50 - 65	50 - 65	50 - 65	-	-	-	-	-
Dietary fiber (g/day)	-	-	-	-	-	≥ 10	≥ 11	≥ 13	≥ 17	≥ 19	-	-	-	-	-
Sodium (g NaCl equivalent/day)	-	-	-	-	-	< 4.5	< 5.0	< 6.0	< 7.0	< 7.5	-	-	-	-	-
Potassium (mg/day)	-	-	-	-	-	≥ 1800	≥ 2000	≥ 2200	≥ 2400	≥ 3000	-	-	-	-	-
Calcium (mg/day)	600	650	700	1000	800	-	-	-	-	-	-	-	-	-	-
Magnesium (mg/day)	130	170	210	290	360	-	-	-	-	-	-	-	-	-	-
Phosphorus (mg/day)	-	-	-	-	-	-	-	-	-	-	-	-	-	-	-
Iron (mg/day)	5.5	7.0	8.5	10.0	10.0	-	-	-	-	-	30	35	35	40	50
Zinc (mg/day)	5	6	7	10	12	-	-	-	-	-	-	-	-	-	-
Copper (mg/day)	0.4	0.5	0.6	0.8	0.9	-	-	-	-	-	-	-	-	-	-
Manganese (mg/day)	-	-	-	-	-	-	-	-	-	-	-	-	-	-	-
Iodine (µg/day)	75	90	110	140	140	-	-	-	-	-	550	700	900	2000	3000
Selenium (µg/day)	15	20	25	30	35	-	-	-	-	-	150	200	250	350	400
Vitamin A (µg RAE/day)	400	500	600	800	900	-	-	-	-	-	950	1200	1500	2100	2500
Vitamin D (µg/day)	-	-	-	-	-	-	-	-	-	-	30	40	60	80	90
Vitamin E (mg/day)	-	-	-	-	-	-	-	-	-	-	300	350	450	650	750
Vitamin K (µg/day)	-	-	-	-	-	-	-	-	-	-	-	-	-	-	-
Thiamin (mg/day)	0.8	1.0	1.2	1.4	1.5	-	-	-	-	-	-	-	-	-	-
Riboflavin (mg/day)	0.9	1.1	1.4	1.6	1.7	-	-	-	-	-	-	-	-	-	-
Niacin (mg/day)	9	11	13	15	17	-	-	-	-	-	100	150	200	250	300
Vitamin B6 (mg/day)	0.8	0.9	1.1	1.4	1.5	-	-	-	-	-	20	25	30	40	50
Vitamin B12 (µg/day)	1.3	1.6	1.9	2.4	2.4	-	-	-	-	-	-	-	-	-	-
Folate (µg/day)	140	160	190	240	240	-	-	-	-	-	400	500	700	900	900
Pantothenic acid (mg/day)	-	-	-	-	-	-	-	-	-	-	-	-	-	-	-
Vitamin C (mg/day)	60	70	85	100	100	-	-	-	-	-	-	-	-	-	-

[a] DRI values are from DRIs for Japanese, 2015.

RDA, Recommended Dietary Allowance; DG, Tentative Dietary Goal for Preventing Lifestyle-related Diseases; UL, tolerable upper intake level; SFA, saturated fatty acid;

PUFA, polyunsaturated fatty acid; 1 µg RAE = sum of retinol (µg) + β-carotene (µg) × 1/12 + α-carotene (µg) × 1/12 + β-cryptoxanthin (µg) × 1/24. 1 g NaCl equivalent = 58.5/23 × sodium (g).
